# Triploid parthenogenetic female *Helophorus brevipalpis* Bedel, 1881 (Coleoptera, Helophoridae) near Oslo, Norway

**DOI:** 10.3897/compcytogen.20.213474

**Published:** 2026-10-07

**Authors:** Robert B. Angus

**Affiliations:** 1 Department of Life Sciences (Insects), The Natural History Museum, London SW7 5BD, UK Department of Life Sciences (Insects), The Natural History Museum London United Kingdom

**Keywords:** C-banding, Coleoptera, *Helophorus brevipalpis*, Norway, Parthenogenesis, triploidy

## Abstract

Triploid female *Helophorus brevipalpis* Bedel, 1881 are recorded from one locality near Oslo, Norway. Analysis of their karyotypes using both conventional Giemsa staining and C-banding reveals a chromosomal arrangement similar to that shown by English diploid material and different from the arrangements shown by known parthenogenetic populations in Spain, France and Italy, indicating that this parthenogenesis has evolved independently.

## Introduction

*Helophorus brevipalpis* Bedel, 1881 is a very common beetle over much of Europe, noted for its swarming in mid- to late summer when it may land in great numbers on shiny surfaces.

The first indication that *H. brevipalpis* could be parthenogenetic came from the discovery of a females-only population in Logan Canyon, Utah, USA (Angus 1971). This, the only genuine North American record of the species, suggested that it had to be an introduction.

Parthenogenetic triploid specimens of *H. brevipalpis* were first identified from Spain, Provincia de León, where they were found along with bisexual diploids (Angus 1992). This record was followed by the discovery of parthenogenetic triploids in the Po valley in Italy (Angus and Foster 2009; Angus and Jia 2020). Then Angus (2025) found triploid material in the Camargue (France). The French and Spanish material has the same chromosomal arrangement, but the Italian population shows minor differences.

In July 2025 Czesław Greń (Bytom, Poland), who already knew of my work on parthenogenetic *H. brevipalpis* (Angus 2025) emailed me to say that he had taken a sample of 126 *Helophorus brevipalpis* near Ski in Norway, of which only 12 of the specimens were males. Acting on this information, accompanied by my daughter Helen Hosier, I visited the area during the first week of July this year and found the material reported here.

## Material and methods

**Figure 1.**
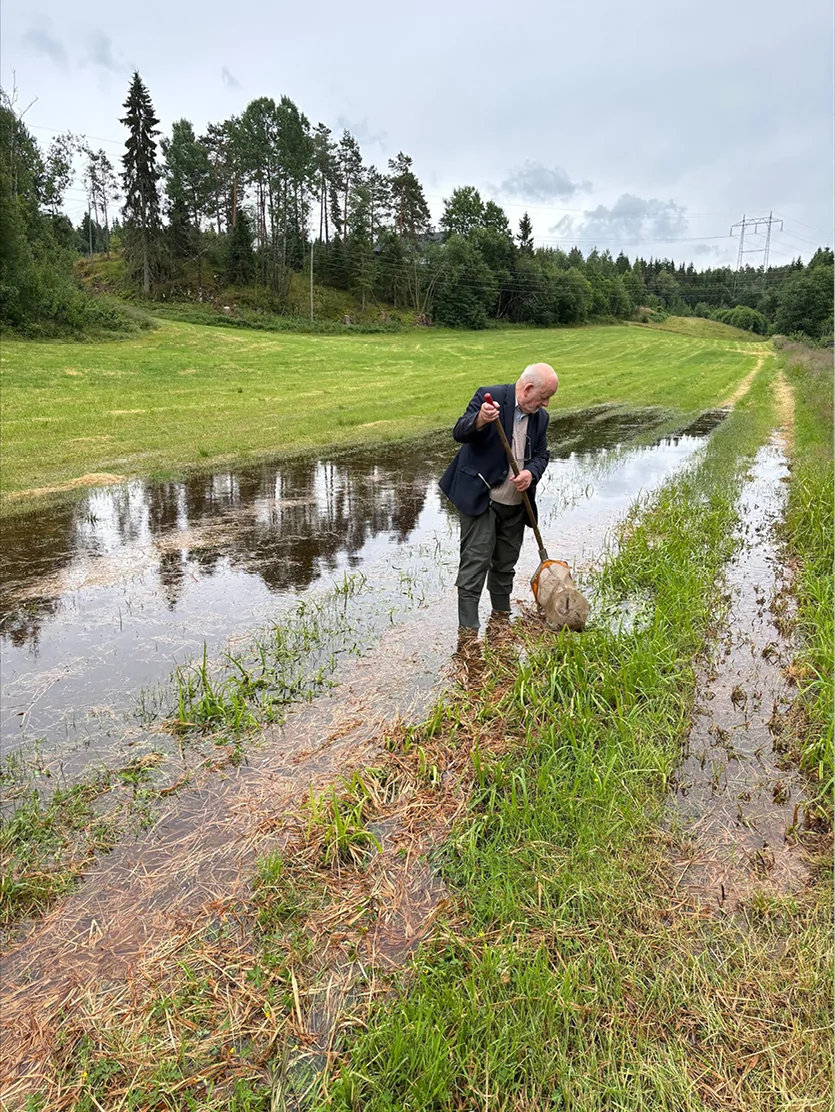
*H. brevipalpis* collecting site rear Ski, Norway. Photo by Helen Hosier.

All the living Norwegian material was collected from the flooded field track west of Ski, Akershus, 59.93988°N, 10.93933°E (Fig. 1). The material was collected on two days, 2^nd^ and 6^th^ July 2026, the first day to find a suitable place and first sample, and the second to collect beetles which would be in transit for as short a time as possible. In all I estimated we collected more than 100 beetles though in the end the retained sample amounted to 69 beetles.

On our return to England on July 7^th^, we found that southeast England was enduring a heatwave with temperatures above 30 °C, so I placed the living material in the salad drawer of a refrigerator. This certainly avoided mortality, but it also resulted an absence of cell-division in the midguts of the beetles. Transfer of the beetles to an aquarium in my garden shed resulted in a resumption of cell division. Beetles were processed for chromosome slides using the methods described by Angus (2025). Beetles were processed on July 8^th^, 9^th^ and 10^th^ (10 specimens per day), and July 17^th^ (9 specimens). The preparations on July 8^th^ and 9^th^ failed, while July 10^th^ gave four preparations indicating triploid females, with a further four on July 17^th^. On July 30^th^ I rounded up all the livestock left in the aquarium, 28 specimens, including one diploid male from which I obtained chromosomes. The 27 females were simply killed by immersion in boiling water and mounted. Chromosome preparations were photographed under oil immersion in the Digitisation and Microscopy laboratory (formerly the Sackler laboratory) in the Natural History Museum, London (NHMUK), using an Olympus BX63 microscope attached to an Olympus DP73 camera. Images were captured using the Olympus cellSens Dimension software (version number 4.2.1), as single optical sections, under brightfield illumination. Whole beetles and genitalia were photographed in the same laboratory, under brightfield illumination, using a Leica 125 stereomicroscope with a Canon 650D camera. EOS Utility (version number 2.14.20.0) was used for image capture and Helicon Focus Pro software (version number 8.3.5) for stacking. All the specimens are now housed in the general collection of the NHMUK.

## Results and discussion

Countable triploid nuclei were obtained from eight of the thirty eight females processed. As mentioned under the listing of material and methods, the first 20 attempted preparations failed, so the success rate once successful preparations were achieved was eight out of 19.

**Figure 2.**
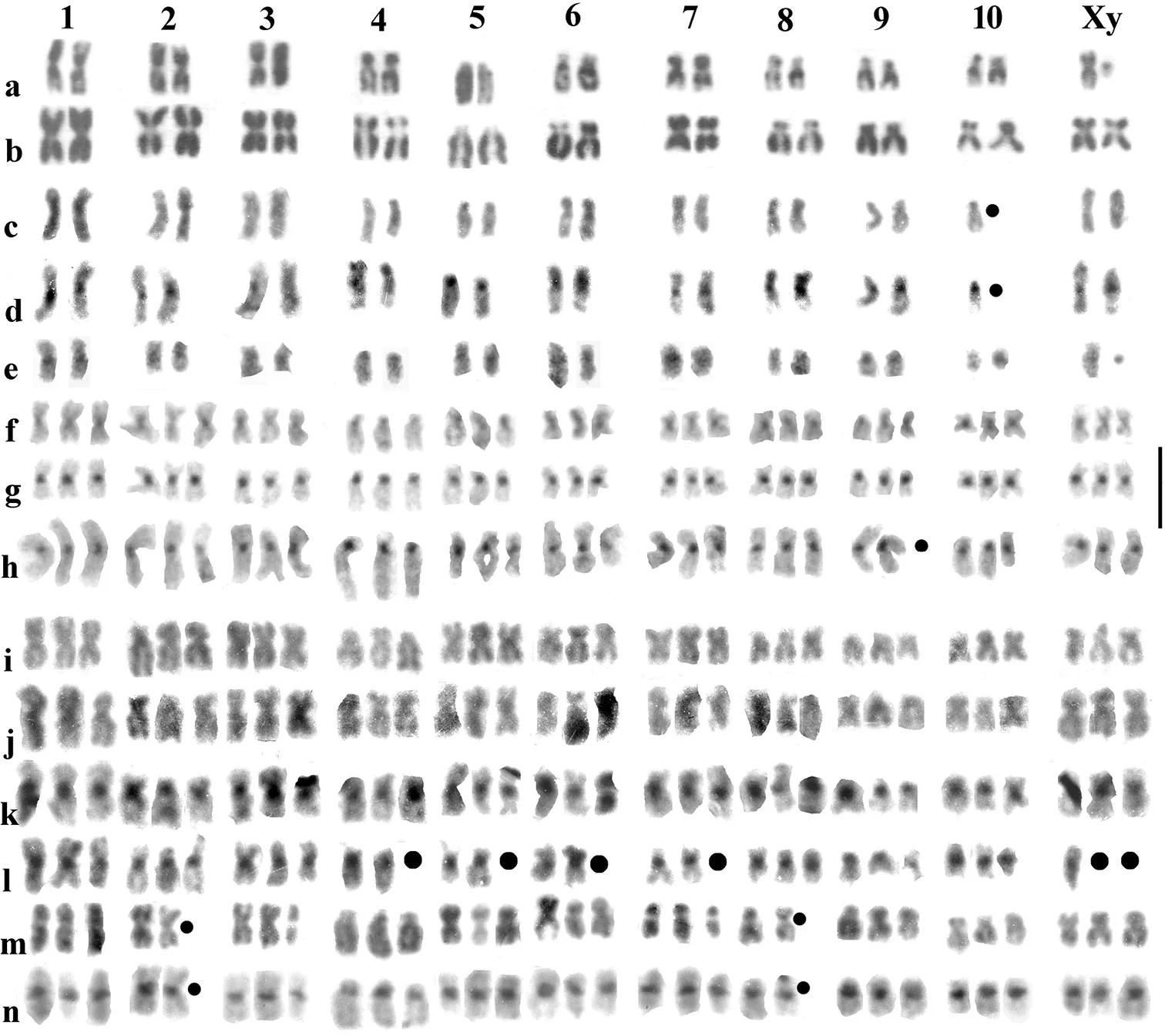
**a–n**. mitotic chromosomes of *Helophorus brevipalpis*, arranged as karyotypes. **a, b**. From embryos; **c–n**. From midgut cells of adult beetles. **a, b**. England, Somerset, Brean, **a**. ♂ **b**. ♀; **c, d**. ♀ England, Hampshire, New Forest, **c**. Giemsa-stained; **d**. The same nucleus C-banded; **e**. ♂ Norway, near Ski, C-banded; **f–n**. Triploid ♀♀. **f, g**. Near Ski; **f**. Giemsa-stained; **g**. The same nucleus C-banded; **h**. Less condensed nucleus, Ski, C-banded; **i**. Spain, León, Algadefe, Giemsa-stained; **j, k, l**. France, Hérault, Salagou River; **j**. Giemsa-stained; **k**. The same nucleus C-banded; **l**. Incomplete nucleus C-banded; **m, n**. Italy, Reggio Emilia, Sologno, from Angus and Jia (2020); **m**. Giemsa-stained; **n**. The same nucleus C-banded. Suggested positions of missing chromosomes indicated by bold dots (•). Scale line: 5 μm.

Karyotypes obtained from Norwegian material are shown in Fig. 2e–h. The arrangement of the chromosomes, in terms of acrocentric versus metacentric, is the same as in English material (Fig. 2a–d). Chromosomes 1–3 are metacentric, 4 and 5 are acrocentric and 6 is submetacentric. Of the smaller chromosomes, 7 to 10 are submetacentric to acrocentric. Acrocentric chromosome 4 is shown by the less condensed preparation (Fig. 2h) to be almost telocentric.

This pattern of chromosome arrangement is clearly different from those shown by published Spanish and French material which has chromosome 2 acrocentric (Angus 1992, 2025), (Fig. 2i–l) as well as that from Italy in which chromosome 5 is metacentric (Angus and Jia 2020) (Fig. 2m, n). For study of the C-banding of the French material I re-examined my original negatives. This clarified the shapes of several of the chromosomes and also showed that during the C-banding treatment some had acquired extraneous heavily staining particles (Fig. 2k, chromosomes 5 and X, one replicate of each triplet). The incomplete C-banded preparation (Fig. 2l) shows the acrocentric chromosome 2 very clearly, with the short arm extended in one replicate. Chromosome 4 is also acrocentric, with the short arm appearing relatively shorter than that of chromosome 2. The Italian material (Fig. 2m, n) shows the longest acrocentric in position 4.

It is thus clear that the known development of parthenogenesis and triploidy has occurred on three separate occasions.

**Figure 3.**
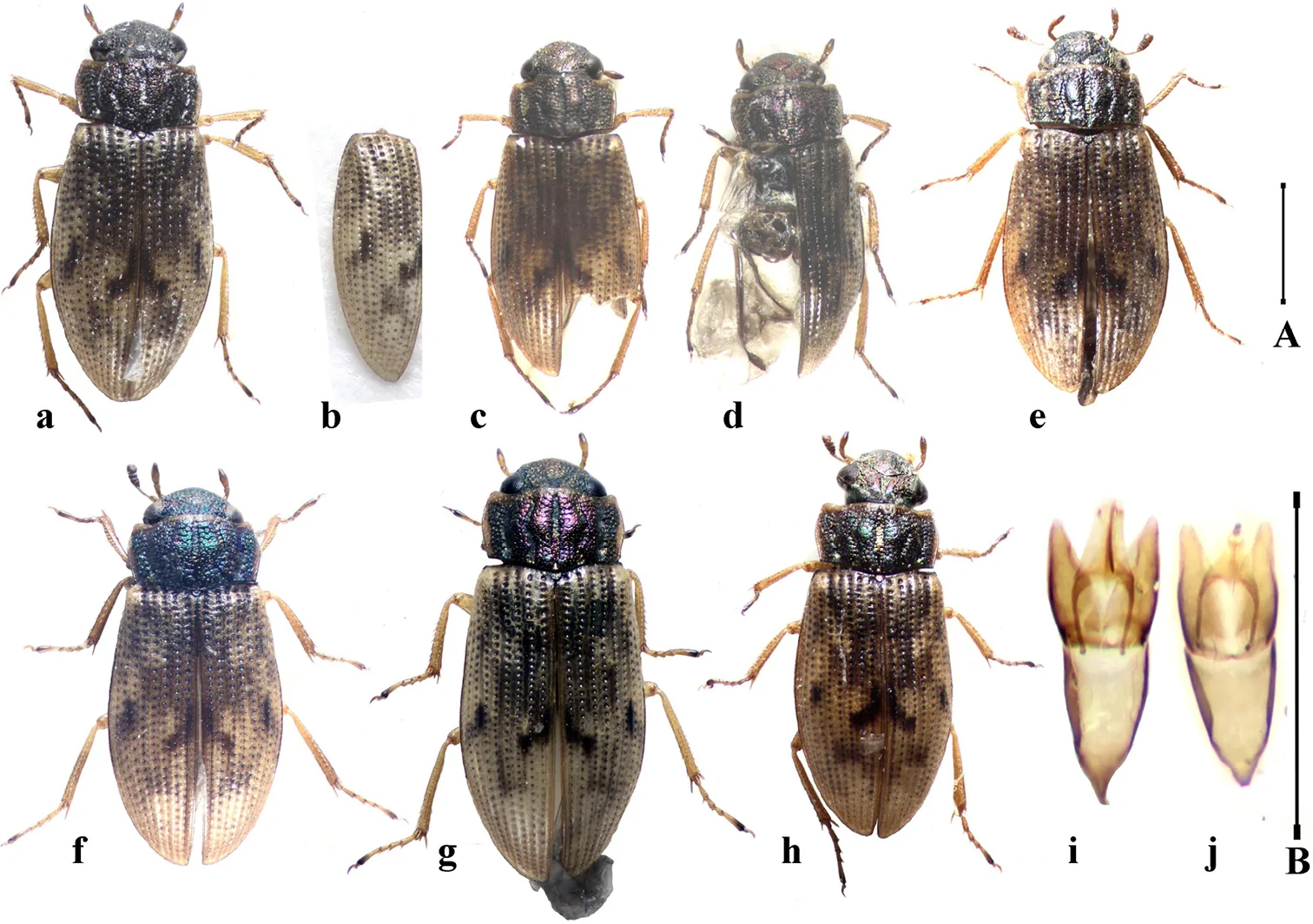
*Helophorus brevipalpis*. **a–h**. Whole beetles; **i, j**. Aedeagi. **a–d**. From near Ski, Norway, **a**. Triploid female; **b**. Detached elytron; **c**. ♂ 1; **d**. ♂ 2; **e**. ♂ from Finland, Karelia australis; **f**. Triploid ♀ from Spain, León. Algadefe; **g**. Triploid ♀ from France, Bouches du Rhone, Camargue; **h**. From Italy, Reggio Emilia, Sologno; **i**. ♂ 1, **j**. ♂ 2. Scale bars: 1 mm (A for **a–h**); 0.5 mm (B for **i, j**).

The Norwegian *H. brevipalpis* (Fig. 3a–d) are noticeably dark, with heavily mottled elytra and the main area of the pronotum, apart from the yellowish anterior and lateral margins is black, sometimes shining but without any greenish- or maroon bronze reflections. This colouration is matched by a male I took in Finland (Parikkala, Karelia australis) in 1970. The aedeagi of the two Norwegian males Fig. 3i, j) as well as the Finnish one, are similar in size to English material, as well as Bedel’s (French) lectotype. The Shetland Island *H. brevipalpis* var. *bulbipalpis* Kuwert, 1890 has a slightly larger aedeagus. Thus the aedeagi of the two Norwegian males are 0.4 and 0.37 mm long (from Fig. 6i, j), the length of the aedeagus of the lectotype of *H. brevipalpis* is 0.45 mm and of *H. brevipalpis* var. *bulbipalpis* is 0.52 mm (Angus 1984, plate 2, figs 50, 54).

## Additional information

### Artificial Intelligence (AI) use

The authors accept full responsibility for the content of the manuscript, including the disclosure of any use of AI.

No AI tools were used in the preparation of this manuscript.

### Author ORCIDs

R.B. Angus https://orcid.org/0000-0002-3860-5617

### Data availability

All of the data that support the findings of this study are available in the main text.

## References

[B1] Angus RB (1971) *Helophorus brevipalpis* Bedel in North America (Coleoptera: Hydrophilidae). The Coleopterists Bulletin 25(4): 129–130. 10.5962/p.372578

[B2] Angus RB (1984) Towards a Revision of the Palaearctic Hydrophiloidea of the genus *Helophorus* F. 1. Entomologicheskoe Obozrenie 63: 533–551[8 plates]. [In Russian]

[B3] Angus RB (1992) A chromosomal investigation of *Helophorus brevipalpis* Bedel (Coleoptera: Hydrophilidae) with triploid Spanish females a possible source of American parthenogenetic material. The Entomologist 111(1): 56–60.

[B4] Angus RB (2025) Triploid female *Helophorus brevipalpis* Bedel, 1881 in Provence, France, with additional data on C-banding in both triploid and diploid material, and discussion of chromosomal variation in *Helophorus brevipalpis*. Comparative Cytogenetics 19: 125–131. 10.3897/compcytogen.19.162685 PMC12311475 40747451

[B5] Angus RB, Foster GN (2009) Italy 24–26 May 2008. Latissimus 25: 4–7.

[B6] Angus RB, Jia F (2020) Triploidy in Chinese parthenogenetic *Helophorus orientalis* Motschulsky, 1860, further data on *H. brevipalpis* Bedel, 1881 and a brief discussion of parthenogenesis in Hydrophiloidea (Coleoptera). Comparative Cytogenetics 14(1): 1–10. 10.3897/CompCytogen.v14i1.47656 PMC6971125 31988701

